# B Cell Deficiency Impairs Collateral Artery Growth by Influencing Early and Late Regenerative Inflammation

**DOI:** 10.3390/cells15151372

**Published:** 2026-07-29

**Authors:** Matthias Kübler, Amanda Geml, Katharina Elbs, Franziska Heim, Kira-Sofie Wimmer, Carolin Baur, Daphne Merkus, Elisabeth Deindl

**Affiliations:** 1 Institute of Surgical Research, LMU University Hospital, LMU Medizin, Ludwig-Maximilians-Universität München, 81377 Munich, Germany; matthias.kuebler@med.uni-muenchen.de (M.K.); amanda.geml@med.uni-muenchen.de (A.G.); katharina.elbs@med.uni-muenchen.de (K.E.); franziska.heim@med.uni-muenchen.de (F.H.); kira.wimmer@med.uni-muenchen.de (K.-S.W.); daphne.merkus@med.uni-muenchen.de (D.M.); 2 Institute of Cardiovascular Physiology and Pathophysiology, Biomedical Center (BMC), LMU Medizin, Ludwig-Maximilians-Universität München, 82152 Planegg-Martinsried, Germany; 3 Department of Oral- and Cranio-Maxillofacial Surgery, Friedrich-Alexander-Universität Erlangen-Nürnberg and Uniklinikum Erlangen, 91054 Erlangen, Germany; 4 Center for Cardiovascular Research (DZHK), Munich Heart Alliance (MHA), Partner Site Munich, 81377 Munich, Germany; 5 Department of Cardiology, German Heart Center, TUM University Hospital, TUM School of Medicine and Health, Technical University of Munich, 80636 Munich, Germany; 6 Department of Experimental Cardiology, Erasmus University Medical Center, 3015 GD Rotterdam, The Netherlands

**Keywords:** arteriogenesis, B lymphocytes, collateral artery growth, macrophage polarization, γδ T cells, mast cells, platelet–neutrophil aggregates, adaptive immunity, hindlimb ischemia, peripheral artery disease

## Abstract

**Highlights:**

- The absence of B cells amplifies early innate immune activation while impairing arteriogenesis.
- B cell deficiency interferes with the temporal γδ T cell reprogramming associated with M2-like macrophage polarization and collateral remodeling.

**Abstract:**

Arteriogenesis, the growth of pre-existing collateral arteries in response to arterial occlusion, is critically regulated by perivascular immune cells. While innate immune contributions are well-established, the role of B lymphocytes remains poorly understood. We investigated the impact of B cell deficiency on arteriogenesis using B cell-deficient J_H_T mice in a murine hindlimb model of femoral artery ligation (FAL). Laser-Doppler perfusion imaging, immunofluorescence staining, Giemsa staining, flow cytometry, and differential blood counts were performed at defined time points after FAL. B cell-deficient mice exhibited significantly impaired perfusion recovery on days 3 and 7 post-FAL, accompanied by reduced collateral artery diameter growth and diminished vascular cell proliferation. Early immune analysis revealed elevated platelet–neutrophil aggregate (PNA) formation and increased perivascular mast cell degranulation in B cell-deficient mice despite unchanged circulating cell counts. On day 7, perivascular M2-like macrophage numbers were selectively reduced. Furthermore, B cell deficiency disrupted the temporal reprogramming of γδ T cell subsets, impairing the shift from pro-inflammatory IFN-γ-associated to anti-inflammatory IL-10-associated subpopulations. These findings demonstrate for the first time that B cells are essential coordinators of the sequential innate and regenerative immune response driving productive collateral artery growth, positioning them as potential targets for therapeutic arteriogenesis strategies.

## 1. Introduction

Cardiovascular diseases (CVDs) remain the leading cause of death worldwide [1]. Narrowing or complete occlusion of arterial vessels results in an inadequate supply of oxygen and nutrients to the distal tissues, causing ischemia and, when prolonged, ischemic tissue damage downstream of the blockage [2]. Depending on the location, this results in coronary heart disease (CHD), cerebrovascular accidents (CVAs), and peripheral artery disease (PAD). Current treatments often depend on invasive interventions, which pose considerable risks, particularly for the high-risk multimorbid patient population. However, the human body possesses the capacity to remodel pre-existing collateral arteries into fully functional conductance vessels in order to compensate for the loss of arterial function caused by occlusion or stenosis [3]. This adaptive vascular remodeling process, referred to as arteriogenesis, represents the growth and maturation of natural bypass vessels [4]. When physiological blood flow through a major artery is compromised, arteriogenesis plays a critical role in maintaining adequate tissue perfusion and preserving organ function. Although it constitutes the only endogenous mechanism capable of functionally compensating for arterial obstruction, the development of effective collateral circulation is a time-consuming and highly regulated process [4]. A comprehensive understanding of the underlying molecular, cellular, and hemodynamic mechanisms governing arteriogenesis is therefore essential for the development of potential non-invasive therapeutic strategies aimed at promoting vascular growth and functional recovery after arterial stenosis or obstruction.

Initially, arterial occlusion results in an increase in shear stress within the pre-existing collateral vessels, resulting in endothelium activation and the initiation of arteriogenesis. As part of the initial mechanosensory signal transduction, the increased shear stress leads to the release of extracellular RNA (eRNA) from endothelial cells [5]. eRNA promotes the initiation of an inflammatory cascade by promoting the binding of vascular endothelial growth factor A (VEGF-A) to vascular endothelial growth factor receptor 2 (VEGFR2). Subsequent VEGFR2 signaling induces the release of von Willebrand Factor (vWF) from Weibel–Palade bodies. vWF then engages the platelet receptor glycoprotein Ib alpha (GPIbα), leading to platelet activation and the formation of platelet–neutrophil aggregates (PNAs) [5]. Activated neutrophils, following extravasation into the perivascular space, stimulate mast cells via the generation of reactive oxygen species (ROS). Mast cell activation is essential for the recruitment of leukocytes, particularly macrophages, to the perivascular tissue [6]. The accumulation of these immune cells enhances the local availability of pro-arteriogenic growth factors, thereby facilitating and amplifying collateral vessel remodeling [7].

Whereas the functions of myeloid cells in arteriogenesis are well-described, little is known about the role of adaptive immune cells. As part of the adaptive immune system, B lymphocytes have various functions. They mediate humoral immune responses and play a crucial role in defending the organism against various pathogens through antigen presentation to T cells, pro-inflammatory cytokine secretion, and antibody production. Beyond this, they have been described to possess antibody-independent functions in sterile inflammation [8,9,10].

A significant interaction between lymphocytes and myeloid cells has already been described in cardiovascular remodeling processes. For instance, after acute myocardial infarction, B cells can recruit monocytes, promoting inflammation and fibrosis [11]. In contrast, interleukin 10 (IL-10)-producing regulatory B cells exert anti-inflammatory effects, thus supporting cardiac remodeling and ameliorating the outcome after acute myocardial infarction [12]. Taken together, these findings highlight the critical role of functional heterogeneity among lymphocyte subsets in determining the outcome of cardiovascular remodeling processes [13].

Current evidence indicates that the adaptive immune system contributes to the pathogenesis of cardiovascular diseases. Recently, we demonstrated that the absence of functional lymphocytes in B cell- and T cell-deficient Rag1-knockout mice resulted in impaired arteriogenesis mirrored by reduced vascular cell proliferation, smaller inner luminal diameter, and altered perivascular macrophage polarization [14].

However, the impact of B lymphocytes on collateral artery growth in particular has yet to be evaluated. Therefore, in this study, we aimed to investigate the effect of the absence of B cells on arteriogenesis in our well-established murine hindlimb model [2].

## 2. Materials and Methods

### 2.1. Animal Protocol

All procedures were approved by the Bavarian Animal Care and Use Committee (ethical approval code: ROB-55.2–2532.Vet_02-17-99, approval date: 8 December 2017; ROB-55.2–2532.Vet_02–22–99, approval date: 29 March 2023) and were conducted in accordance with Directive 2010/63/EU of the European Parliament on the protection of animals used for scientific purposes.

Experiments included B cell-deficient B6.129P2-Igh-J^tm1Cgn^/J mice (hereafter referred to as J_H_T mice or B cell −/− mice) (*n* = 5 per time point), kindly provided by Prof Dr. Klaus Rajewsky (Max Delbrück-Center, Berlin, Germany) and bred at our institute [15,16]. B cell deficiency was confirmed by genotyping (see below). Wildtype (WT) C57BL/6J mice (*n* = 5 per time point) were obtained by Charles River Laboratory (Sulzfeld, Germany). Mice were housed under standard laboratory conditions with ad libitum access to enrichment, food, and water.

Male B cell −/− mice (8–12 weeks old) were subjected to femoral artery ligation (FAL) and compared to age- and sex-matched WT mice. All experimental procedures were performed in accordance with ARRIVE, institutional, and national guidelines.

### 2.2. Experimental Procedures

Anesthesia was induced via subcutaneous (s.c.) injection of a triple combination consisting of midazolam (5.0 mg/kg, Ratiopharm GmbH, Ulm, Germany), fentanyl (0.05 mg/kg, CuraMED Pharma, Karlsruhe, Germany), and medetomidine (0.5 mg/kg, Pfister Pharma, Berlin, Germany) (hereafter abbreviated MMF). After ensuring adequate anesthesia, the right femoral artery was ligated distal to the A. femoralis profunda, while a sham operation was performed on the contralateral side as an internal control as previously described [2]. The femoral artery ligation led to the induction of arteriogenic growth of collateral arteries in the adductor muscle. Mice were maintained on a 37 °C warming pad throughout surgery.

To label proliferating cells, mice received daily intraperitoneal (i.p.) injections of Bromodeoxyuridine (BrdU; 1.25 mg dissolved in phosphate-buffered saline (PBS), Sigma-Aldrich, St. Louis, MO, USA), beginning directly after the surgical intervention and continuing throughout the observation period.

For measurements of perfusion recovery on day 3 after FAL, the animals were anaesthetized by continuous administration of 1.5% isoflurane. In all other instances, the animals were anaesthetized by subcutaneous injection of MMF as described before.

Adductor muscles and whole-blood samples were isolated on day 1 (*n* = 5 per group), day 3 (*n* = 5 per group), and day 7 (*n* = 5 per group) after FAL.

### 2.3. Perfusion Assessment by Laser-Doppler Imaging

Perfusion of the hindlimbs was quantified longitudinally using a Laser-Doppler imaging system (Moor LDI 5061, Moor Instruments, Axminster, UK; software version 3.01). Measurements were obtained prior to surgery, directly following arterial ligation, confirming sufficient ligation of the femoral artery, and again on days 3 and 7 postoperatively.

To minimize the impact of external influences such as warmer ambient temperature and possible vasoconstricting effects of the used anesthetics, the measurements were carried out over an acclimatization period of 10 min for each mouse. An area of exactly 0.42 cm^2^ was defined on each paw, while the perfusion of the sham-operated side was set as the reference value to which the percentages of the operated side referred.

### 2.4. Tissue and Blood Collection

For harvesting the adductor muscles, mice were re-anesthetized as described above and sacrificed by cervical dislocation. Tissue perfusion was performed via aortic catheterization, first flushing with an adenosine buffer (5% bovine serum albumin (BSA, Sigma-Aldrich) and 1% adenosine (Sigma-Aldrich, dissolved in PBS), followed by in situ fixation using 3% paraformaldehyde (PFA, Merck, Darmstadt, Germany; dissolved in PBS). Tissues were fixed in Tissue-Tek compound (Sakura Finetek Germany GmbH, Staufen, Germany). The tissues were stored at −80 °C for further investigation. Whole blood was collected via cardiac puncture and heparinized for further flow cytometry and differential blood analyses.

### 2.5. Immunohistology

Cryosections (8 µm) of the adductor muscle were prepared for histological and immunohistochemical analysis. For all tissue analyses employing immunofluorescence staining, 3 tissue sections with 2 (growing) collaterals each were evaluated per muscle sample. Depending on the cell population or parameter assessed, either the collateral vessel itself or the immediate surrounding perivascular space was analyzed. Nuclear DNA was visualized in all sections by counterstaining the prepared slides with DAPI (62248, Thermo Fisher Scientific; dilution 1:1000 in PBS/0.1% Tween-20 (AppliChem GmbH) (PBS-T)). The slides were mounted with Dako mounting medium (Agilent, Santa Clara, CA, USA).

Imaging was performed with an epifluorescence microscope (DM6 B, Leica Microsystems, Wetzlar, Germany) using a 20× objective lens (630 μm × 475 μm). Fiji software (ImageJ, Version 1.54p) was used for image processing and quantitative cell analysis.

#### 2.5.1. CD31/ACTA2/BrdU/DAPI Staining

CD31/ACTA2/BrdU/DAPI quadruple immunofluorescence staining was implemented for analyzing vessel diameters and vascular cell proliferation. Initially, tissue sections were incubated with pre-heated 1 M HCl at 37 °C for 30 min and subsequently permeabilized for 10 min with 0.2% Triton X-100 (AppliChem GmbH, Darmstadt, Germany; diluted in PBS/0.5% BSA/0.1% Tween-20) at room temperature (RT). The sections were then blocked at RT for 1 h with 10% goat serum and incubated at 4 °C overnight with the anti-BrdU primary antibody (ab6326, Abcam, Cambridge, UK; rat monoclonal antibody; dilution 1:100 in 10% goat serum). On the following day, tissue sections were stained with the secondary antibody goat-anti-rat (Alexa Fluor 546, A11081, Thermo Fisher Scientific, Waltham, MA, USA; dilution 1:100 in PBS-T) and incubated for 1 h at RT. Afterwards, the sections were blocked for 30 min at RT with 4% BSA (diluted in PBS-T). To label endothelial cells, the sections were counterstained with an anti-CD31 antibody (Alexa Fluor 647, 102516, BioLegend, San Diego, CA, USA; dilution 1:100 in PBS-T), and with an anti-ACTA2 antibody to mark smooth muscle cells (alpha smooth muscle actin 2; Alexa Fluor 488 antibody, F3777, Sigma-Aldrich; dilution 1:400 in PBS-T), followed by incubation for 2 h at RT.

#### 2.5.2. CD31/CD68/MRC1 Staining

To quantify perivascular macrophages and determine their polarization state, tissue sections were fixed in 4% PFA for 10 min at RT, followed by a blocking step with 10% donkey serum for 1 h at RT. The sections were then incubated overnight at 4 °C with the primary antibody anti-MRC1 (mannose receptor C-type 1; ab64693, Abcam; rabbit polyclonal antibody; dilution 1:200 in 10% donkey serum). On the following day, the sections were treated with the secondary antibody donkey-anti-rabbit (Alexa Fluor 546, A-10040, Thermo Fisher Scientific; dilution 1:200 in PBS-T) and incubated for 1 h at RT. A second blocking step was performed using 4% BSA in PBS-T for 30 min at RT. Then, sections were incubated overnight at 4 °C with the antibodies anti-CD68 (Alexa Fluor 488, ab201844, Abcam; dilution 1:200 in PBS-T) and anti-CD31 (Alexa Fluor 647, 102516, BioLegend; 1:100 in PBS-T).

### 2.6. Giemsa Staining

Giemsa staining was performed to visualize perivascular mast cells as well as degranulated perivascular mast cells. For this purpose, tissue sections were fixed in 4% PFA for 5 min, then stained with Giemsa solution (Carl Roth GmbH, Karlsruhe, Germany; 1:25 in distilled water, 1 h at 65 °C), rinsed in 0.5% acetic acid, dehydrated in graded ethanol (96%, 99%), and cleared in xylene. Images were acquired at 40× magnification. For mast cell quantification, 3 sections with 2 collaterals each were evaluated per muscle sample.

### 2.7. Flow Cytometry

Whole blood was collected on day 1 and day 3 after FAL by cardiac puncture into heparinized syringes. Lysis of red blood cells was conducted using BD FACS™ Lysing Solution (BD Biosciences, Franklin Lakes, NJ, USA). Leukocytes and platelets were then pelleted by centrifugation for 5 min at RT at 400× *g*. Sample analysis was conducted on a BD LSRFortessa™ Cell Analyzer (BD Biosciences), and compensation controls were included for each fluorochrome. Data were analyzed using the FlowJo v10 software (BD Biosciences).

#### 2.7.1. Platelet Aggregate Analysis

For quantification of platelet aggregates, cells were incubated with anti-CD11b (PE, 1:300, BioLegend), anti-CD115 (BV421, 1:300, BioLegend), anti-CD41 (FITC, 1:400, BioLegend), anti-Gr-1 (APC, 1:800, BioLegend), and Viability Dye (APC-Cy7, 1:1000, Invitrogen, Waltham, MA, USA) in 1% BSA/PBS for 20 min at 4 °C in the dark, then washed and resuspended in 300 µL 1% BSA for acquisition.

#### 2.7.2. γδ T Cell Subset Analysis

Cells were stained with anti-CD3 (eFluor450, 1:300, Invitrogen), anti-TCRγ/δ (APC, 1:300, BioLegend), anti-CD39 (PE, 1:100, BioLegend), anti-CD27 (FITC, 1:200, BioLegend), and Viability Dye (APC-Cy7, 1:1000, Invitrogen) in 4% BSA/PBS for 20 min at 4 °C in the dark, then washed and resuspended in 300 µL 1% BSA for acquisition.

### 2.8. Differential Blood Analysis

To analyze a possible effect of B cell deficiency on platelet, neutrophil, and monocyte counts in peripheral blood after femoral artery ligation, differential blood counts were performed using an automated hematology analyzer (ProCyte Dx, Idexx Laboratories, Westbrook, ME, USA) with mouse-specific settings. Blood was collected via cardiac puncture from anesthetized mice at 24 h after FAL prior to euthanasia by cervical dislocation.

### 2.9. Genotyping

To confirm B cell deficiency, genotyping was performed according to the protocol of the Jackson Laboratory for the B6.129P2-Igh-J^tm1Cgn^/J strain [16,17]. Genomic DNA was isolated from ear biopsies using the KAPA Express Extract kit (KE7102, Kapa Biosystems, Wilmington, MA, USA), applied according to standard manufacturer protocols, and amplified via two separate PCR reactions distinguishing wildtype and J_H_T alleles. PCR products were separated on a 1.5% agarose gel with a 100 bp DNA ladder.

### 2.10. Statistical Analyses

All statistical evaluations and graphical representations were generated in GraphPad Prism 10 (GraphPad Software, La Jolla, CA, USA). Results are expressed as mean ± standard error of the mean (S.E.M.), which estimates the variability of the group mean. Statistical significance was defined as *p* < 0.05. To ensure transparency and reproducibility, the corresponding figure legends specify the statistical test used for each dataset. The normality of the data was assessed using the Shapiro–Wilk test before performing further statistical analyses.

### 2.11. Illustration

The illustrations from the Graphical Abstract were designed and rendered with BioRender.com (Created in BioRender. Kübler, M. (2026) https://BioRender.com/0pm0d44, accessed on 23 July 2026).

## 3. Results

### 3.1. B Cell Deficiency Impairs Perfusion Recovery After Femoral Artery Ligation

To investigate the effect of the absence of B cells on arteriogenesis, we used a defined murine hindlimb model for collateral vessel growth induction [2]. Hereby, arteriogenesis is induced by unilateral ligation of the right murine femoral artery, leading to collateral artery growth in the adductor muscle of the upper leg. Serving as an internal control, the left femoral artery of the upper leg is sham-operated.

**Figure 1:**
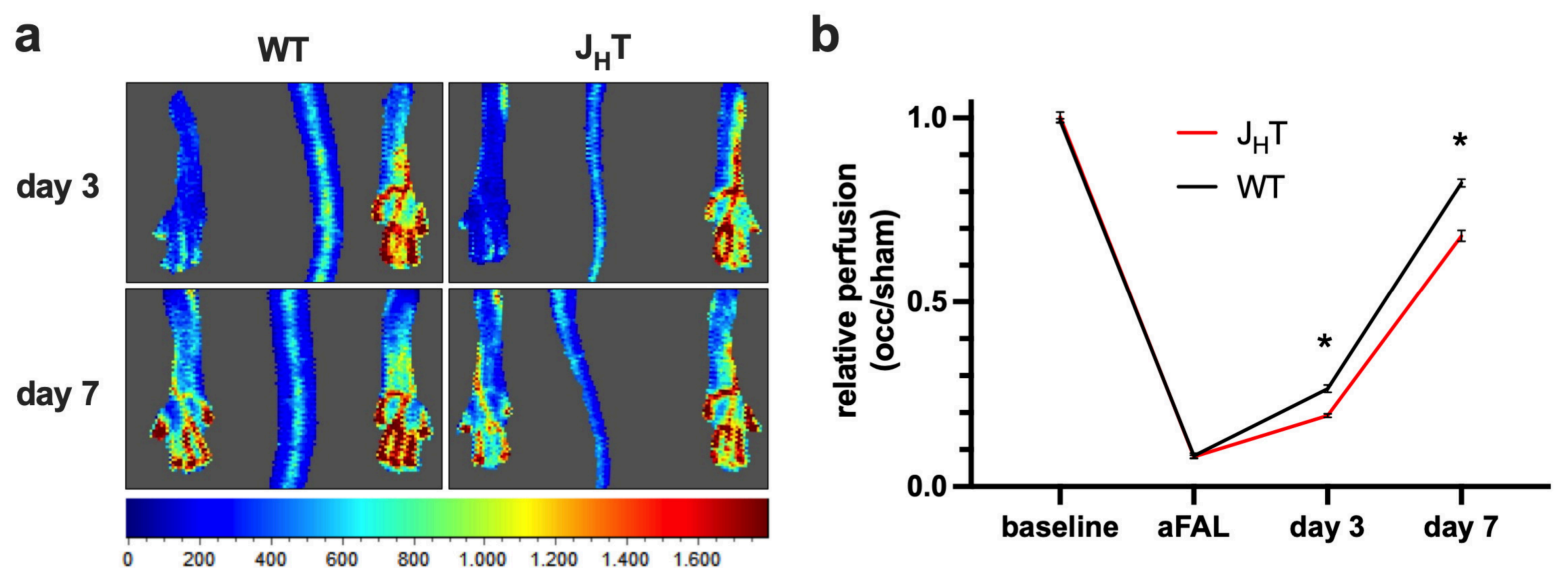
Perfusion recovery after femoral artery ligation (FAL) is decreased in B cell-deficient mice. (**a**) Representative flux images from laser-Doppler imaging (LDI) show the murine hindlimb 3 and 7 days after FAL in wildtype control (WT) and B cell-deficient J_H_T mice. The color scale reflects relative perfusion levels ranging from low (blue) to high (red). (**b**) The graph depicts the relative perfusion (calculated as the ratio of blood flow in the limb distal to the occlusion to the contralateral sham side) after induction of arteriogenesis by FAL in WT control (black line) or in B cell-deficient J_H_T mice (red line) directly prior to (baseline) and immediately after FAL (aFAL) and on days 3 and 7 after FAL as assessed by LDI. Statistical analysis was conducted with two-way ANOVA followed by Bonferroni correction (*n* = 5 per group). Data are means ± S.E.M. * *p* < 0.05 (day 3: WT vs. J_H_T (*p* < 0.05); day 7: WT vs. J_H_T (*p* < 0.05)).

To ascertain the influence of B cell absence on the perfusion recovery after femoral artery ligation (FAL), we performed laser-Doppler perfusion measurements of the murine hindlimbs at defined time points (before (baseline), directly after, and 3 and 7 days after ligation). J_H_T mice (B cell-deficient mice), compared to wildtype mice (WT, control), showed significantly decreased perfusion recovery on day 3 as well as on day 7 after FAL (Figure 1a,b).

### 3.2. The Absence of B Cells Reduces Vessel Diameter Growth and Vascular Cell Proliferation

**Figure 2:**
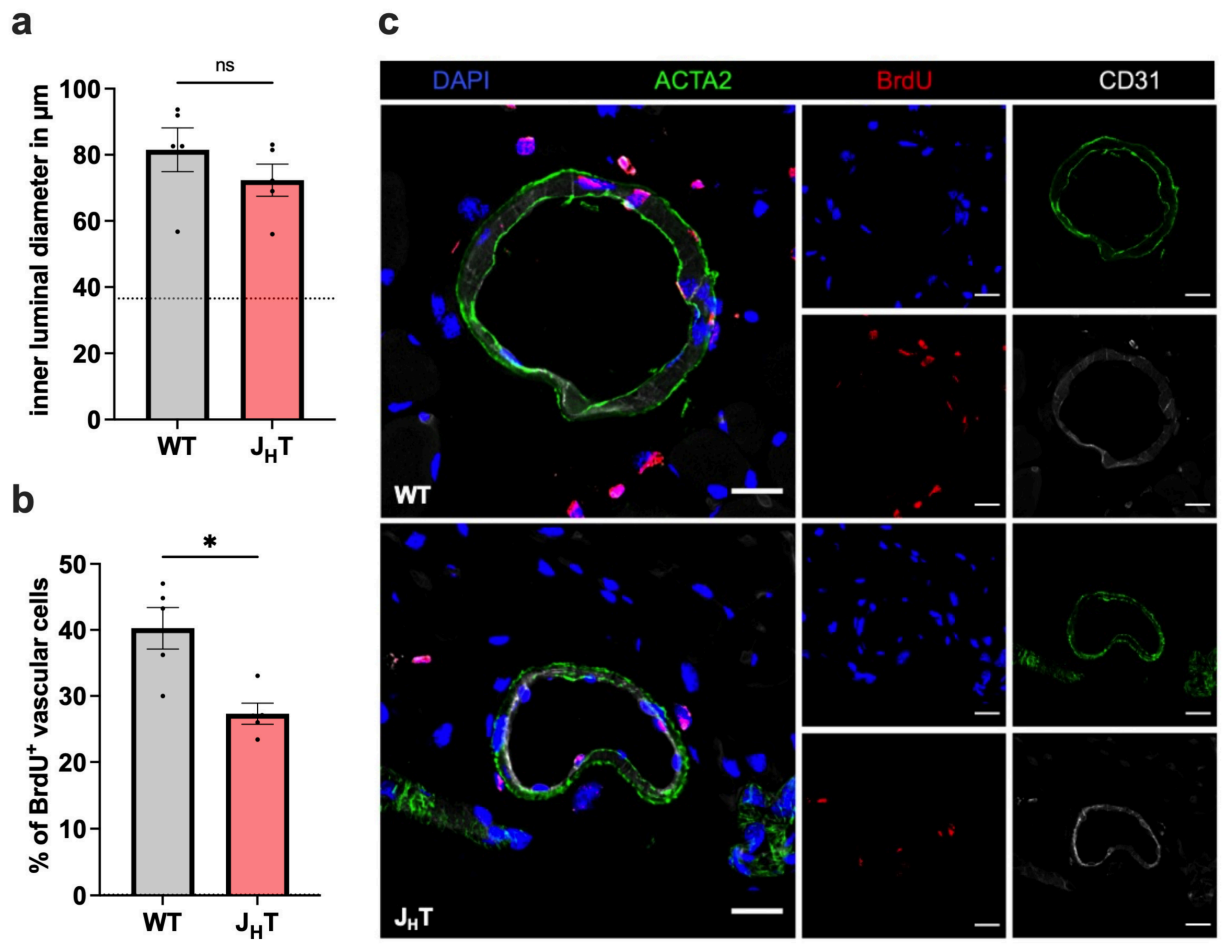
B cell deficiency decreases inner collateral artery diameters and vascular cell proliferation. The bar graphs depict (**a**) the inner luminal diameter in µm and (**b**) the proportion of proliferating (BrdU^+^) vascular cells relative to all vascular cells in wildtype control (WT) and B cell-deficient J_H_T mice 7 days after femoral artery ligation (FAL). The dashed horizontal line represents the mean sham value. Statistical analysis was performed using an unpaired *t*-test (*n* = 5 mice per group; 3 histological sections per mouse, each containing 2 collateral vessels). Data are means ± S.E.M.; * *p* < 0.05, ns = non-significant. (**c**) Representative immunofluorescence images of collateral arteries harvested from WT and J_H_T mice on day 7 after FAL are shown. Cell nuclei are visualized by DAPI (blue), smooth muscle cells are stained with anti-alpha smooth muscle actin 2 (ACTA2, green), endothelial cells are stained with anti-CD31 (white), and proliferating cells are identified by bromodeoxyuridine (BrdU) incorporation (red). Scale bars represent 20 µm.

To validate that the decreased perfusion recovery observed in B cell-deficient mice reflected decreased collateral artery growth, we performed immunofluorescence analyses of the adductor muscle collected 7 days after FAL. A CD31-antibody was implemented as an endothelial cell marker, staining the inner vascular layer, for analysis of the inner luminal diameter. In comparison to the control group, B cell-deficient J_H_T mice showed a reduction in inner luminal diameter (Figure 2a,c). To prove whether this decrease in inner luminal diameter was due to reduced vascular cell proliferation, and not to vasocontraction, combined immunofluorescent staining with bromodeoxyuridine (BrdU) as a cell proliferation marker and alpha smooth muscle actin 2 (ACTA2) as a marker for the outer vessel wall (staining vascular smooth muscle cells) were performed. Consequently, BrdU-positive cells within the vascular structure were defined as proliferating vascular cells (proliferating endothelial cells (Brdu^+^CD31^+^) and proliferating vascular smooth muscle cells (Brdu^+^ACTA2^+^)). We observed a significant reduction in the percentage of proliferating vascular cells in B cell-deficient mice compared to the control group (Figure 2b,c). Analyses of the sham-operated upper hindlimbs showed no significant differences for both inner luminal diameter (WT 39.95 μm ± 5.372 μm vs. J_H_T 33.67 μm ± 3.93 μm, *p* > 0.05) and proliferating vascular cells (WT 0.06667 ± 0.06667 vs. J_H_T 0 ± 0, *p* > 0.05) between wildtype and B cell-deficient mice.

### 3.3. B Cell Deficiency Increases Platelet–Neutrophil Aggregate Formation

**Figure 3:**
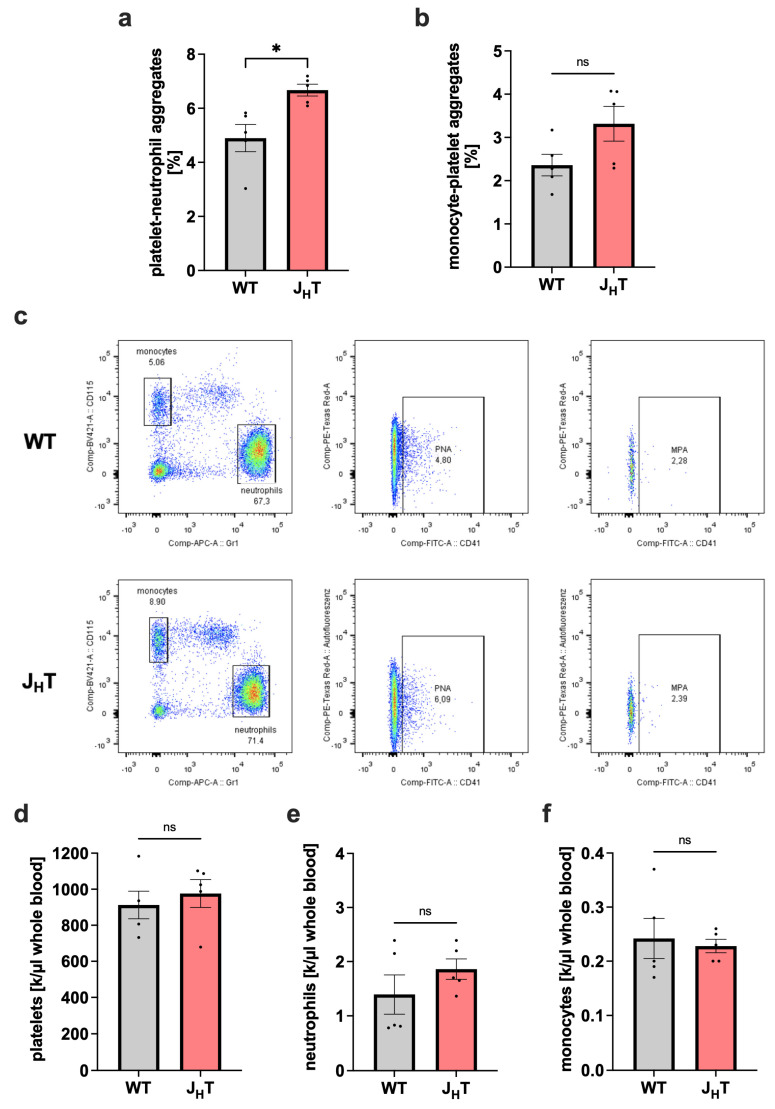
B cell deficiency boosts platelet–neutrophil aggregate (PNA) formation during arteriogenesis. The bar graphs display the percentage of (**a**) PNAs relative to the total number of neutrophils and (**b**) the percentage of monocyte–platelet aggregates (MPAs) relative to the total number of monocytes of wildtype control (WT) and B cell-deficient J_H_T mice 24 h after femoral artery ligation (FAL). (**c**) Representative flow cytometry dot plots showing the gating strategy. Platelets were detected by an anti-CD41 (FITC) antibody, neutrophils were identified by anti-CD11b (PE) and anti-Gr-1 (APC) antibodies, and monocytes by anti-CD11b (PE) and anti-CD115 (Brilliant Violet (BV) 421) antibodies. The bar graphs show the number of (**d**) platelets, (**e**) neutrophils, and (**f**) monocytes per microliter of whole blood measured by differential blood count of WT and J_H_T mice 24 h after FAL. Statistical analysis was performed using an unpaired *t*-test (*n* = 5 per group). Data are means ± S.E.M.; * *p* < 0.05, ns = non-significant.

In the early phases of collateral artery growth, increased shear stress within the collateral vessels leads to platelet activation and their association with circulating leukocytes [6,18]. To quantify these key interactions in the initial stages of arteriogenic growth, we performed flow cytometric profiling of whole-blood samples harvested 24 h after FAL. Compared to wildtype controls, B cell-deficient J_H_T mice exhibited a significant increase in neutrophil–platelet interaction, quantified as CD41^+^/GR-1^+^ platelet–neutrophil aggregates (PNAs), while showing no significant differences in the proportion of monocytes engaging with platelets, identified as CD41^+^/CD115^+^ monocyte–platelet aggregates (MPAs) (Figure 3a–c). To determine whether these findings were attributable to alterations in circulating blood cell counts, we conducted differential blood analyses on whole-blood samples collected 24 h after FAL. B cell deficiency did not significantly affect total platelet, neutrophil, or monocyte counts compared to control mice (Figure 3d–f), indicating that the observed increase in PNAs was not secondary to changes in peripheral cell abundance.

### 3.4. B Cell Deficiency Boosts Perivascular Mast Cell Degranulation

**Figure 4:**
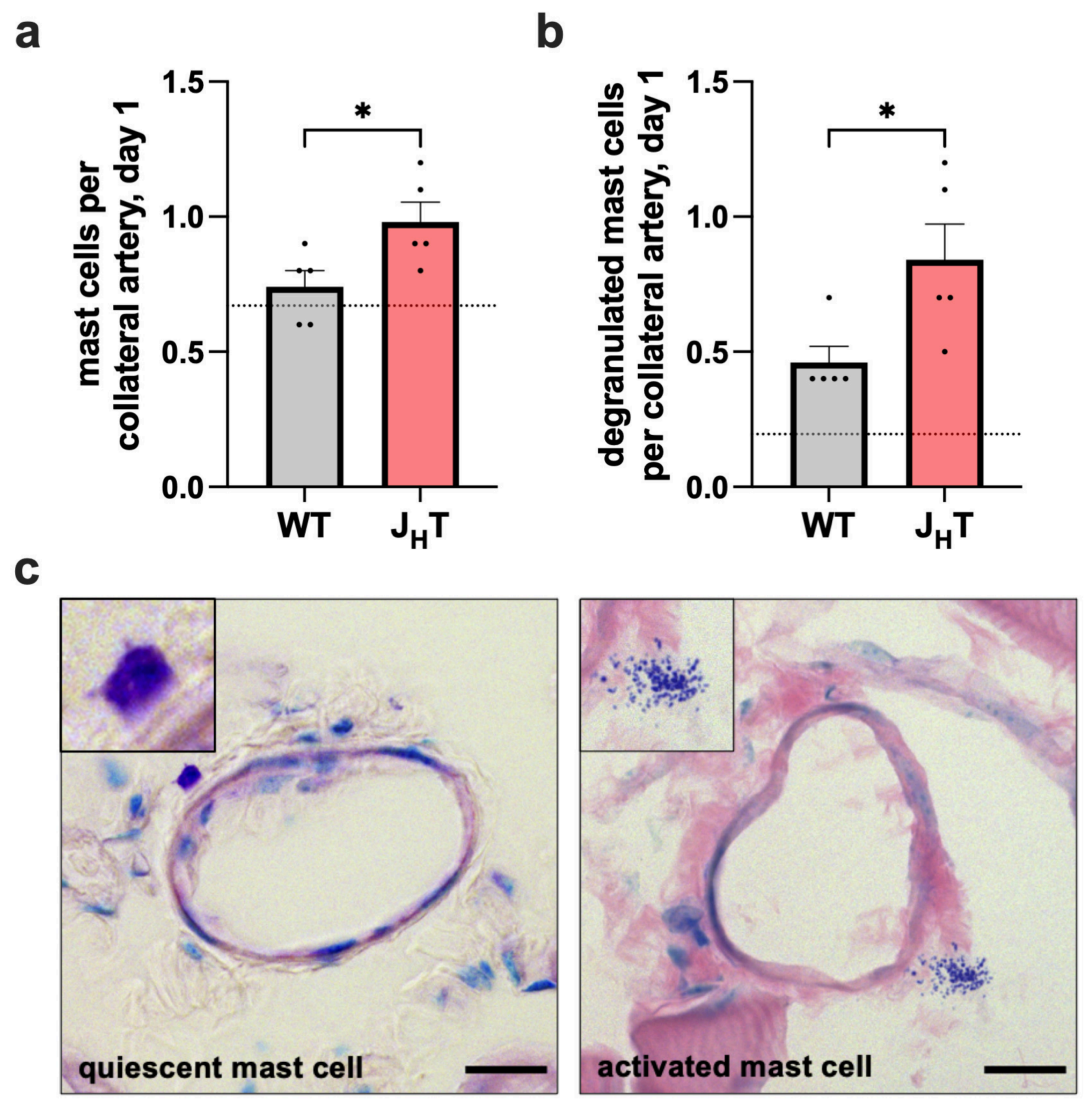
B cell deficiency increases perivascular mast cell accumulation and degranulation in the early phases of arteriogenesis. The bar graphs depict (**a**) the number of perivascular mast cells, quantified as mast cells per collateral artery, as well as (**b**) the number of degranulated mast cells per collateral artery, in wildtype controls (WT) compared with B cell-deficient J_H_T mice. The analysis was performed on Giemsa-stained adductor muscle sections collected on day 1 after femoral artery ligation (FAL). Only mast cells in direct proximity to the growing collateral arteries were counted. The dotted horizontal line indicates the mean mast cell count observed in the contralateral sham-operated side. Statistical analysis was performed using an unpaired *t*-test (*n* = 5 mice per group; 3 histological sections per mouse, each containing 2 collateral vessels). Data are means ± S.E.M.; * *p* < 0.05. (**c**) Representative Giemsa-stained images demonstrate perivascular quiescent (left) and activated (degranulated, right) mast cells around a growing collateral artery 1 day after FAL. Inserts show magnified images of representative mast cells. Scale bars represent 20 μm.

The recruitment and activation of mast cells in the perivascular space plays a pivotal role in the regulation of arteriogenesis [6]. The activation of mast cells in the perivascular space of collaterals depends on platelet activation via platelet receptor GPIbα, followed by PNA formation [18]. To analyze whether B cell deficiency affects mast cell dynamics during arteriogenic bypass growth, we performed Giemsa staining on collateral arteries harvested 1 day after FAL. We found significantly elevated numbers of total perivascular mast cells as well as degranulated (hence activated) perivascular mast cells on day 1 after FAL in J_H_T mice compared to control mice (Figure 4). Analyzing the sham-operated tissues, we did not find any significant differences in total (WT 0.6667 ± 0.1667 vs. J_H_T 0.68 ± 0.01, *p* > 0.05) or degranulated perivascular mast cell count (WT 0.05667 ± 0.5667 vs. J_H_T 0 ± 0, *p* > 0.05) between wildtype and B cell-deficient mice.

### 3.5. The Absence of B Cells Leads to Decreased Macrophage Accumulation and Altered Macrophage Polarization in the Perivascular Space at Late Inflammatory Stages

Macrophages and their state of polarization play an important role in the regulation of the processes of arteriogenesis [19,20]. Hence, we analyzed perivascular macrophage accumulation as well as their polarization state 7 days after FAL by immunofluorescence staining. We implemented an anti-CD68 antibody as a marker for macrophages and an anti-mannose receptor c-type 1 (MRC1) antibody as a marker for the M2-like polarized phenotype. Thus, CD68^+^MRC1^−^ cells were counted as pro-inflammatory M1-like polarized macrophages, whereas CD68^+^MRC1^+^ cells were quantified as anti-inflammatory and regenerative M2-like polarized macrophages.

**Figure 5:**
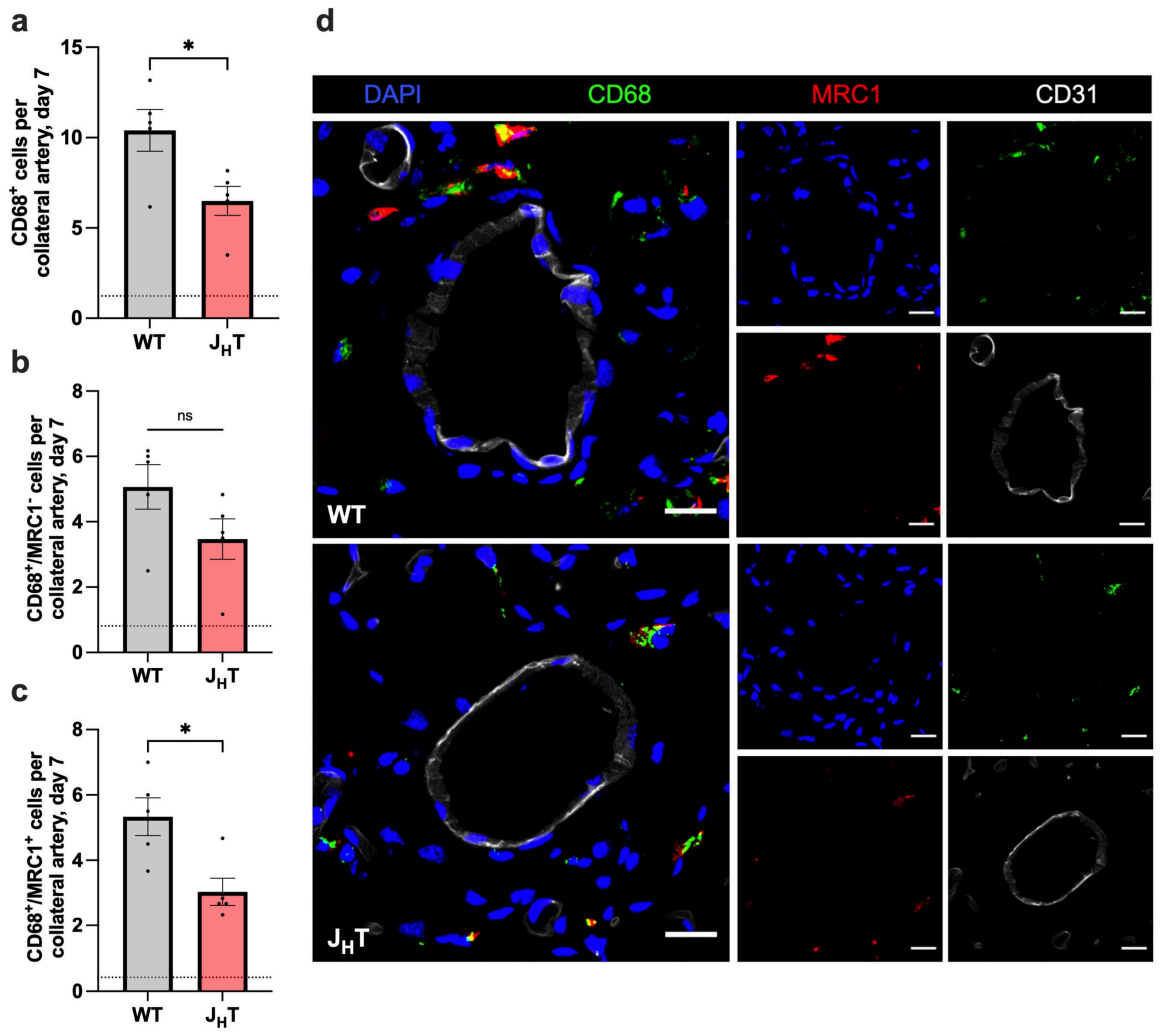
B cell deficiency interferes with macrophage recruitment and polarization. The bar graphs depict (**a**) the total number of macrophages (CD68^+^) per collateral artery, (**b**) the number of M1-like polarized macrophages (CD68^+^/MRC1^−^ (mannose receptor C type 1)) per collateral artery, (**c**) and M2-like polarized macrophages (CD68^+^/MRC1^+^) per collateral artery in wildtype control (WT) and B cell-deficient J_H_T mice on day 7 after femoral artery ligation (FAL). The dotted line represents the average value of the sham-operated side. Statistical analysis was conducted with an unpaired *t*-test (*n* = 5 per group; 3 histological sections per mouse, each containing 2 collateral vessels). Data are means ± S.E.M.; * *p* < 0.05, ns = non-significant. Only macrophages in the perivascular space up to a distance that excluded skeletal muscle fibers were quantified. (**d**) Representative immunofluorescence staining of collateral arteries (endothelial cells, anti-CD31, white) and perivascular macrophages (anti-CD68, green) with M1-like (MRC1^−^) or M2-like (anti-MRC1^+^, red) polarization in WT (upper panels) and J_H_T mice (lower panels) on day 7 after FAL. The larger images show the merging of the smaller single-channel images. Scale bars represent 20 µm.

In tissue harvested 7 days after FAL, we observed a significantly decreased number of perivascular macrophages per growing collateral in B cell-deficient mice compared to wildtype mice (Figure 5a,d). The reduction was due to reduced numbers of M2-like polarized macrophages, while no significant difference was observed in numbers of M1-like polarized macrophages (Figure 5b–d). Sham-operated tissues showed no significant differences regarding total macrophage (WT 1.277 ± 0.2936 vs. J_H_T 1.167 ± 0.1934, *p* > 0.05), M1-like polarized (WT 0.9433 ± 0.529 vs. J_H_T 0.61 ± 0.28, *p* > 0.05), or M2-like polarized (WT 0.39 ± 0.3089 vs. J_H_T 0.5567 ± 0.3389, *p* > 0.05) cell count.

### 3.6. B Cell Deficiency Influences Temporal Shifts in γδ T Cell Subsets

Recently, we showed that γδ T cells, a small T cell fraction, play an important role in influencing arteriogenesis by contributing sequentially to both the inflammatory initiation and resolution phases of arteriogenesis [21]. During the early phase, pro-inflammatory interferon gamma (IFN-γ)-associated CD27^+^CD39^−^ γδ T cell subsets promote mast cell degranulation. At later stages, anti-inflammatory IL-10-associated CD27^−^CD39^+^ γδ T cell and CD27^+^CD39^+^ regulatory γδ T cell populations modulate macrophage polarization by skewing differentiation toward a pro-arteriogenic M2-like polarized phenotype.

To determine whether B cell deficiency interferes with this temporal shift in γδ T cell subset dynamics, we performed flow cytometric profiling of whole-blood samples harvested on day 1 and day 3 after FAL in J_H_T and WT control mice.

**Figure 6:**
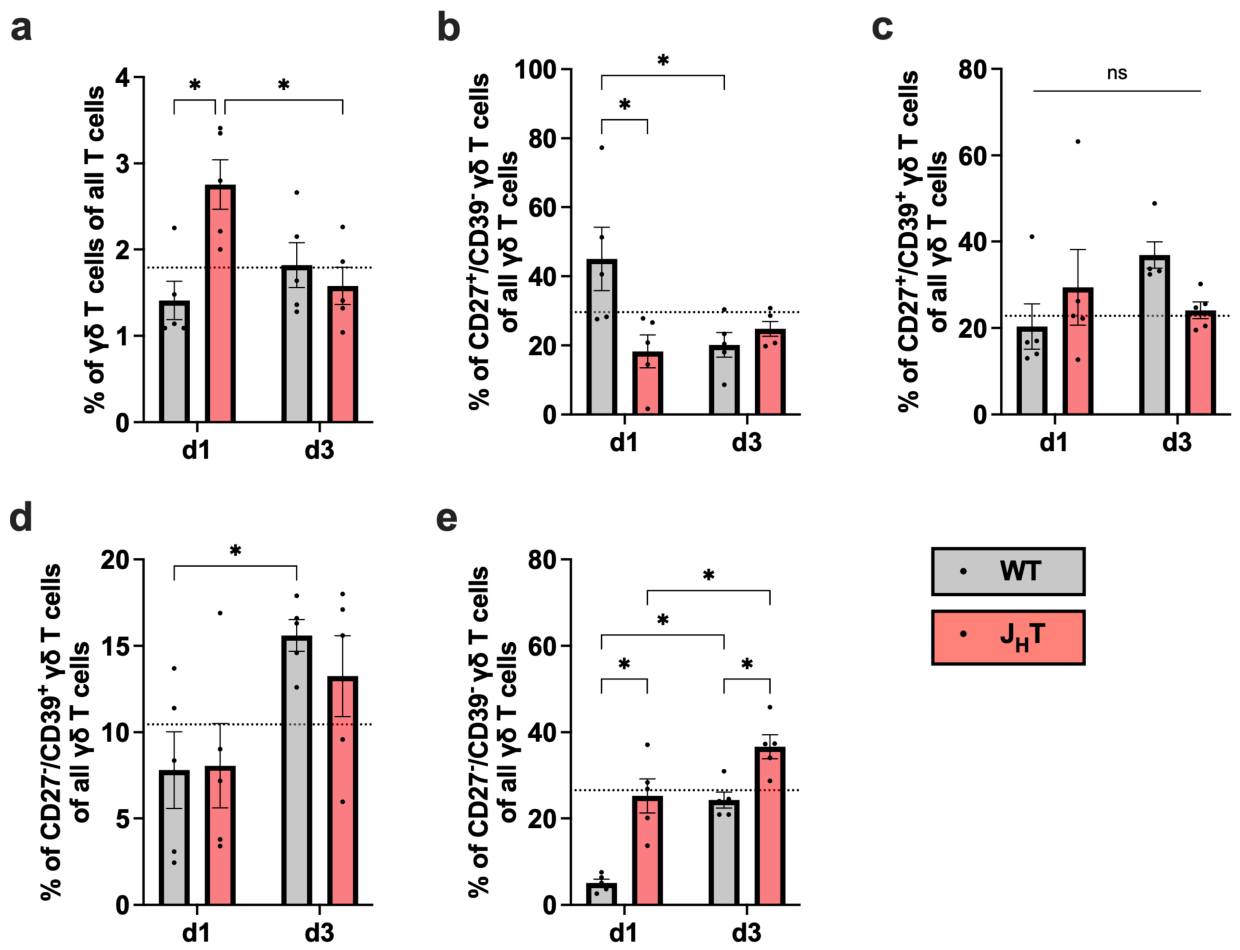
B cell deficiency interferes with temporal γδ T cell subset reprogramming during arteriogenesis. The scatter plots show the percentage of (**a**) γδ T cells relative to all T cells in peripheral blood as well as the percentage of (**b**) IFN-γ-associated CD27^+^CD39^−^ γδ T cells, (**c**) CD27^+^CD39^+^ regulatory γδ T cells, (**d**) IL-10-associated CD27^−^CD39^+^ γδ T cells, and (**e**) double-negative inactive CD27^−^CD39^−^ γδ T relative to all γδ T cells in wildtype (WT) and B cell-deficient J_H_T mice on day 1 and day 3 after femoral artery ligation (FAL). The dotted line represents average cell percentages in sham-operated WT mice. Statistical analysis was conducted with two-way ANOVA (*n* = 5 per group). Data are means ± S.E.M.; * *p* < 0.05, ns = non-significant.

The proportion of γδ T cells within the total T cell compartment was significantly increased in J_H_T mice on day 1, whereas no difference compared with WT controls was observed on day 3. Notably, this proportion declined significantly from day 1 to day 3 in B cell-deficient mice (Figure 6a).

Compared to controls, we observed a significant decrease in the frequency of pro-inflammatory IFN-γ-associated CD27^+^CD39^−^ γδ T cells in B cell-deficient mice on day 1, while no difference between both groups was detected on day 3. Over time, control mice exhibited a significant decrease in this γδ T cell subset from day 1 to 3 after FAL, whereas no significant temporal change was observed in J_H_T mice (Figure 6b).

The proportion of anti-inflammatory regulatory CD27^+^CD39^+^ γδ T cells remained comparable between groups at both time points and did not exhibit significant temporal changes (Figure 6c).

For anti-inflammatory IL-10-associated CD27^−^CD39^+^ γδ T cells, we observed no difference between both groups at either time point. However, we detected a significant increase from day 1 to day 3 in control mice, while B cell-deficient mice exhibited only a non-significant upward trend (Figure 6d).

Analyzing the number of inactive γδ T cells, we found significantly increased numbers in B cell-deficient mice at both day 1 and day 3 after FAL compared to WT controls. Furthermore, both groups demonstrated a significant increase in this subset over time (Figure 6e).

## 4. Discussion

The contribution of the adaptive immune system, specifically B cells, to arteriogenesis is not fully understood. In this study, we demonstrate that B cell deficiency impairs collateral artery growth after femoral artery ligation, highlighting a critical role for B cells in orchestrating arteriogenic growth. We observe that the absence of B cells interferes with both early and late inflammatory responses to arterial occlusion, ultimately leading to the inhibition of collateral artery cell proliferation, resulting in the limited increase in collateral artery diameters and reduced perfusion recovery.

Arteriogenesis constitutes a compensatory vascular growth program initiated in response to arterial occlusion or high-grade stenosis, thereby preserving tissue perfusion and limiting ischemic injury [4]. This process is initially triggered by increased fluid shear stress within pre-existing collateral arterioles and is sustained by leukocyte-derived growth factors and cytokines that orchestrate vascular remodeling [5,6]. Among perivascular infiltrating leukocyte populations, neutrophils, mast cells, and macrophages have been identified as critical mediators of collateral artery growth [6]. Consequently, investigations to date have predominantly focused on the contribution of innate immune cell subsets.

B cells represent the sole hematopoietic cell population capable of differentiating into antibody-secreting cells, also referred to as plasma cells. Beyond their role in humoral immunity, B cells function as antigen-presenting cells and are potent producers of immunomodulatory cytokines, thus modulating the function of macrophages, T cells, and other leukocytes [9,22]. However, little is known about their role in arteriogenesis. Recently, we demonstrated that B cell- and T cell-deficient Rag1-KO mice showed impaired arteriogenic growth, thus suggesting an important role of the adaptive immune system modulating the processes of natural bypass formation [14]. For the first time, in the present study, we show that the absence of B cells alone interferes with perfusion recovery after FAL and hence collateral artery growth, confirmed by laser-Doppler perfusion measurements. Furthermore, our immunofluorescence stains clearly show that the decreased perfusion recovery in B cell-deficient mice is attributed to decreased inner luminal diameter and reduced numbers of proliferating endothelial and vascular smooth muscle cells in collateral arteries compared to WT controls. A direct effect of B cells on proliferating vasculature cells via secretion of VEGF-A has been described in the context of angiogenesis and lymphangiogenesis in lymph nodes [23]. However, in the context of arteriogenesis, bioavailability of VEGF-A is not the limiting factor in collateral artery growth [24]. We propose that B cells primarily influence the proliferation of vascular endothelial cells and smooth muscle cells indirectly via immunomodulatory effects and the control of other immune cells.

To understand the mechanistic basis of impaired arteriogenesis in B cell-deficient mice, we examined the early immune response on day 1 after FAL. As already mentioned, the processes of arteriogenesis are initiated by increased shear stress, leading to endothelial cell activation and the subsequent release of vWF [5,25]. vWF then activates platelets, which results in leukocyte–platelet interactions, in particular PNA and MPA formation. While platelet-activated monocytes extravasate into the perivascular space and differentiate into macrophages, PNA formation is a prerequisite for mast cell activation via neutrophil-derived ROS [6,26,27]. In the present study, we demonstrate that B cell deficiency significantly upregulates the formation of PNAs after the induction of arteriogenesis without altering platelet or neutrophil whole-blood counts. It has been described that lymphocytes can influence platelet activation as well as conjugation with other platelets and leukocytes [28,29]. However, a direct effect of B cells or B cell deficiency on PNA formation has not yet been described. We propose an indirect effect of B cell deficiency leading to ameliorated PNA formation, possibly via changes in cytokine secretion, like the absence of regulatory B cells (Breg)-associated IL-10, affecting integrin MAC-1 (CD11b/CD18) expression on neutrophils, that acts as key receptor for PNA formation [18,30]. Further experiments focusing on the expression of MAC-1 during arteriogenesis in B cell deficiency need to elucidate this possible pathway.

Not only accumulation but the concomitant degranulation of perivascular mast cells is known to be a crucial step in arteriogenesis, ultimately leading to the recruitment of perivascular macrophages [21]. As described earlier, mast cell degranulation depends on ROS derived from platelet-activated and extravasated neutrophils. Consistent with the elevated numbers of PNAs, we observed increased levels of degranulated perivascular mast cells in B cell-deficient mice. Additionally, J_H_T mice showed increased total numbers of perivascular mast cells, independent of their activation state. We hypothesize that the absence of B cells triggers a compensatory immune response, resulting in an excess of PNA formation and consequently driving both the enhanced recruitment and activation of perivascular mast cells. Supporting this notion, Bregs have been described to suppress mast cell activation via IL-10-dependent mechanisms in the context of IgE-mediated allergic reactions, suggesting that the loss of this Breg-mediated inhibitory axis in J_H_T mice may further amplify mast cell activation [31]. However, further experimental approaches are necessary to investigate this B cell–mast cell interactions in the setting of collateral artery growth.

Interestingly, both ameliorated PNA formation as well as subsequent enhanced mast cell degranulation are associated with increased perfusion recovery via promoting early perivascular leukocyte recruitment [6]. However, despite harboring elevated numbers of (activated) mast cells, B cell-deficient J_H_T mice show significantly impaired perfusion recovery as early as day 3 after FAL. We hypothesize that B cell-deficient mice develop an early hyperinflammatory response that, rather than promoting productive arteriogenic remodeling, results in insufficient arteriogenesis. This is reflected, in part, by the observed hyperstimulation of mast cells. To further understand the early changes in the arteriogenesis-associated inflammation in J_H_T mice, we analyzed circulating γδ T cell populations on day 1 and day 3 after FAL. γδ T cells have recently been identified as sequential modulators of arteriogenesis: pro-inflammatory IFN-γ-associated CD27^+^CD39^−^ γδ T cells promote mast cell degranulation in the early phase, while anti-inflammatory CD27^−^CD39^+^ and regulatory CD27^+^CD39^+^ γδ T cell subsets modulate macrophage polarization toward the pro-arteriogenic M2-like phenotype at later stages [21]. Our data reveal a diminished proportion of pro-inflammatory CD27^+^CD39^−^ γδ T cells in J_H_T mice on day 1, despite elevated mast cell activation at this point. This dissociation suggests that the augmented early mast cell response in B cell-deficient animals is not driven by γδ T cell-mediated IFN-γ signaling but may instead reflect compensatory overactivation of mast cells through enhanced PNA formation. Beyond its role in leukocyte recruitment, mast cell degranulation is also known to promote arteriogenesis through the activation of matrix metalloproteinases (MMPs), which facilitate perivascular extracellular matrix remodeling required for collateral vessel expansion [6,32,33]. We propose that, under physiological conditions, this process depends on a tightly controlled, temporally and spatially restricted release of proteases and other granule contents. An excessive, dysregulated mast cell activation, as observed in B cell-deficient mice, may disrupt this balance, shifting the outcome from a locally defined, productive matrix remodeling process toward a diffuse, uncontrolled proteolytic and oxidative tissue insult. Rather than facilitating structured vascular wall reorganization, such excessive activation could thus contribute to perivascular tissue damage that impairs, rather than supports, collateral artery growth. In line with this, a previous study demonstrated that B cell deficiency alone, in the absence of any surgical intervention, is sufficient to induce endothelial dysfunction as well as vascular oxidative stress and inflammation [34]. A comparable underlying pro-inflammatory predisposition could similarly account for the enhanced perivascular mast cell infiltration and degranulation observed in our model, which may thus rather mirror a generalized pro-inflammatory state than reflect a productive, pro-arteriogenic mast cell activation. Taken together, these considerations suggest that the increased mast cell activity observed in B cell-deficient mice may be insufficient to initiate pro-arteriogenic signaling, and may instead represent an excessive inflammatory reaction in the absence of regulatory B cell subsets, such as Bregs. Additionally, further analyses focusing on the local concentrations of pro-inflammatory markers, such as IFN-γ, are necessary to substantiate these hypotheses.

Notably, total γδ T cell frequency within the T cell compartment was significantly elevated on day 1 in J_H_T mice but declined significantly by day 3—a temporal dynamic not observed in WT control mice. This transient expansion followed by a subsequent reduction in the γδ T cell pool may reflect compensatory or reactive mobilization in the absence of B cells, though its functional significance requires further investigation. The increased proportion of inactive γδ T cells in J_H_T mice at both time points suggests that, despite their numerical expansion, γδ T cells in B cell-deficient animals are functionally suppressed—pointing to a possible role for B cells in affecting γδ T cell effector activity.

Mast cell activation ultimately results in an increased permeability of the endothelial barrier and subsequently in the enhanced perivascular infiltration of further leukocyte populations, especially monocytes [6,35]. Once recruited to the perivascular site, monocytes differentiate into macrophages, which secrete pro-arteriogenic cytokines, growth factors, and matrix-remodeling enzymes. These perivascular macrophages play an essential role in arteriogenic growth, as they stimulate the proliferation of vascular endothelial and smooth muscle cells [20,36,37]. By day 7, J_H_T mice displayed a significantly reduced total number of perivascular macrophages, attributable exclusively to a reduction in M2-like polarized macrophages, while M1-like numbers remained unchanged. We obtained similar observations in our previous study on arteriogenesis in B cell and T cell deficiency [14]. M2-like polarized macrophages are central mediators of the resolution phase of arteriogenesis, secreting growth factors including VEGF-A, platelet-derived growth factor subunit B (PDGF-B), and transforming growth factor beta (TGF-β), which support vascular smooth muscle cell proliferation and extracellular matrix remodeling [6,37]. Their selective reduction in B cell-deficient mice at a time point corresponding to the peak of collateral diameter growth strongly implicates impaired M2-like polarization as a key mechanism underlying the impaired perfusion recovery observed. B cells have been shown to possess immunosuppressive functions [38]. Additionally, it was recently reported that B cells, especially regulatory B cells, can promote anti-inflammatory macrophage polarization [39,40,41]. Hence, we propose that B cells may support M2-like polarization either directly via secretion of IL-10 or interleukin 4 (IL-4), or indirectly by influencing the bioavailability of anti-inflammatory γδ T cell populations that in turn modulate macrophage phenotype [21]. The γδ T cell data provide a potential mechanistic link: the failure of CD27^−^CD39^+^ IL-10-associated γδ T cells to significantly expand from day 1 to day 3 in J_H_T mice, while controls showed a significant increase, may translate into reduced IL-10-mediated M2 polarization signals reaching macrophages in the collateral environment at later time points.

The γδ T cell dynamics on day 3 further support this model. In wildtype controls, a significant temporal shift occurred between day 1 and day 3: pro-inflammatory CD27^+^CD39^−^ γδ T cells declined, while anti-inflammatory CD27^−^CD39^+^ γδ T cells increased—consistent with a programmed transition from the inflammatory initiation phase to the pro-arteriogenic resolution phase [21]. In J_H_T mice, neither of these temporal shifts reached statistical significance, indicating that B cell deficiency disrupts the coordinated temporal reprogramming of the γδ T cell compartment. This impaired transition likely contributes to the failure to sustain anti-inflammatory macrophage polarization on day 7. It is noteworthy that the regulatory CD27^+^CD39^+^ γδ T cell subset remained comparable between groups at both time points, suggesting that this regulatory population is not the primary mediator of B cell-dependent γδ T cell modulation in arteriogenesis.

Summarizing these findings, we propose the following role for B cells in arteriogenesis: in wildtype mice, B cells coordinate the temporal transition from the pro-inflammatory initiation phase to the pro-arteriogenic resolution phase of arteriogenesis. During the early inflammatory phase, B cells—possibly acting through Bregs—appear to calibrate the magnitude of PNA formation and mast cell degranulation, as their absence is associated with a compensatory overshooting of both processes that does not lead to sufficient arteriogenic growth. Additionally, B cells support the temporal shift of γδ T cells from an IFN-γ-associated to an IL-10-associated phenotype, thereby facilitating M2-like macrophage polarization in the perivascular space. The absence of B cells results in dysregulated early innate immune activation (increased PNA formation and mast cell degranulation), followed by impaired resolution (failure of γδ T cell reprogramming and consequent M2 macrophage deficit), ultimately manifesting as reduced collateral diameter growth and poor perfusion recovery. This model positions B cells as immune coordinators rather than direct effectors in arteriogenesis, consistent with emerging evidence that B cells, especially regulatory B cells, shape tissue immune responses through cytokine production, antigen presentation, and modulation of other lymphocyte populations [9,10,42].

Several limitations of the present study merit discussion. First, the use of germline B cell-deficient J_H_T mice precludes distinguishing developmental compensatory effects from the acute absence of B cells during arteriogenesis. Second, the mechanistic link between B cells and macrophage polarization inferred from our data remains correlative. The present study establishes that global B cell absence affects collateral artery growth, without resolving the contribution of individual, functionally distinct B cell subpopulations to this process. Functional rescue experiments—such as adoptive transfer of specific, defined B cell subsets, like Bregs, into B cell-deficient J_H_T mice, or B cell-specific genetic manipulation of IL-10 signaling—would be required to establish causality and identify the functionally relevant B cell population. In this context, it is important to note that regulatory B cells do not represent a fixed developmental lineage but rather a functional, context-dependent cellular state, and their IL-10-producing phenotype may not be stably maintained following adoptive transfer into a new microenvironment. Hence, genetic approaches targeting IL-10 signaling directly in B cells may represent a more robust complementary strategy to dissect this pathway. Moreover, the analyses of which specific Breg subsets are functionally relevant in the context of arteriogenesis is still unclear and needs to be elucidated in further in-depth studies. Third, our analyses were restricted to selected time points; more granular temporal profiling may reveal additional phases of B cell involvement. Future studies should also address whether B cell-derived antibodies contribute to arteriogenesis independently of cellular immune coordination, and whether the B cell-dependent mechanisms identified here are conserved in pathological contexts such as peripheral artery disease in aged or dyslipidemic animals. Finally, our findings are derived from a murine model of arteriogenesis, and no clinical correlates or outcome measurements were assessed. While the observed differences in perfusion recovery between wildtype and B cell-deficient mice were statistically significant, their translational and clinical relevance to PAD in humans remains to be established in future studies.

In conclusion, this study demonstrates for the first time that B cells are required for adequate arteriogenesis following femoral artery ligation. B cell deficiency leads to a paradoxical early immune hyperactivation—characterized by increased PNA formation and mast cell degranulation—followed by failure of the pro-arteriogenic resolution phase, marked by impaired γδ T cell reprogramming and selective loss of M2-like macrophage polarization on day 7. These findings reveal B cells as essential coordinators of the sequential immune response that drives productive collateral artery growth, and open new avenues for investigating B cell-targeted strategies in the context of therapeutic arteriogenesis.

## Data Availability

The data presented in this study are available on request from the first author.

## Abbreviations

The following abbreviations are used in this manuscript:

ACTA2	alpha smooth muscle actin 2
BrdU	bromodeoxyuridine
Bregs	regulatory B cells
BSA	bovine serum albumin
CHD	coronary heart disease
CVAs	cerebrovascular accidents
CVDs	cardiovascular diseases
DNA	deoxyribonucleic acid
eRNA	extracellular RNA
FAL	femoral artery ligation
γδ T cell	gamma delta T cell
GPIbα	glycoprotein Ib alpha
IFN-γ	interferon gamma
IL-4	interleukin 4
IL-10	interleukin 10
i.p.	intraperitoneal
LDI	laser-Doppler imaging
MMF	midazolam, medetomidine, fentanyl
MMP	matrix metalloproteinase
MPA	monocyte–platelet aggregate
MRC1	mannose receptor C-type 1
ns	non-significant
PAD	peripheral artery disease
PBS	phosphate-buffered saline
PDGF-B	platelet-derived growth factor subunit B
PFA	paraformaldehyde
PNA	platelet–neutrophil aggregate
ROS	reactive oxygen species
RT	room temperature
s.c.	subcutaneous
S.E.M.	standard error of the mean
TGF-β	transforming growth factor beta
VEGF-A	vascular endothelial growth factor A
VEGFR2	vascular endothelial growth receptor 2
vWF	von Willebrand Factor
WT	wildtype
